# Degradable 2-Hydroxyethyl Methacrylate/Gelatin/Alginate Hydrogels Infused by Nanocolloidal Graphene Oxide as Promising Drug Delivery and Scaffolding Biomaterials

**DOI:** 10.3390/gels8010022

**Published:** 2021-12-27

**Authors:** Marija M. Babić Radić, Vuk V. Filipović, Marija Vukomanović, Jasmina Nikodinović Runić, Simonida Lj. Tomić

**Affiliations:** 1 University of Belgrade, Faculty of Technology and Metallurgy, Karnegijeva 4, 11000 Belgrade, Serbia; 2 University of Belgrade, Institute for Chemistry, Technology and Metallurgy, Njegoševa 12, 11000 Belgrade, Serbia; vukan87@yahoo.com; 3 Advanced Materials Department, Jožef Stefan Institute, Jamova Cesta 39, 1000 Ljubljana, Slovenia; marija.vukomanovic@ijs.si; 4 University of Belgrade, Institute of Molecular Genetics and Genetic Engineering, Vojvode Stepe 444a, 11000 Belgrade, Serbia; jasmina.nikodinovic@imgge.bg.ac.rs

**Keywords:** scaffolding biomaterials, graphene oxide infused hydrogels, alginate/gelatin based hydrogels, curcumin release

## Abstract

The design and evaluation of novel 2-hydroxyethyl methacrylate/gelatin/alginate/graphene oxide hydrogels as innovative scaffolding biomaterials, which concurrently are the suitable drug delivery carrier, was proposed. The hydrogels were prepared by the adapted porogen leaching method; this is also the first time this method has been used to incorporate nanocolloidal graphene oxide through the hydrogel and simultaneously form porous structures. The effects of a material’s composition on its chemical, morphological, mechanical, and swelling properties, as well as on cell viability and in vitro degradation, were assessed using Fourier transform infrared spectroscopy (FTIR), scanning electron microscopy (SEM), measurements of Young’s modulus, gravimetric method and MTT test, respectively. The engineered hydrogels show good swelling capacity, fully hydrophilic surfaces, tunable porosity (from 56 to 76%) and mechanical properties (from 1.69 to 4.78 MPa), curcumin entrapment efficiency above 99% and excellent curcumin release performances. In vitro cytotoxicity on healthy human fibroblast (MRC5 cells) by MTT test reveal that the materials are nontoxic and biocompatible, proposing novel hydrogels for in vivo clinical evaluation to optimize tissue regeneration treatments by coupling the hydrogels with cells and different active agents to create material/biofactor hybrids with new levels of biofunctionality.

## 1. Introduction

Since its first report in the 1960s by Wichterle and Lim, hydrogels are increasingly being used as attractive and promising candidates for multifunctional biomedical uses in the fields of tissue engineering, drug delivery systems, biosensors, and so on, by virtue of their unique structural and compositional similarities to the native extracellular matrix (ECM), three-dimensional polymeric networks of interconnected pores that support cell attachment, migration and proliferation [1]. Plenty of materials, including natural (gelatin, collagen, elastin, alginate, chitosan, fibrin) and synthetic macromolecules poly(vinyl alcohol), polyacrylamide, polypeptide, 2-hydroxyethyl (meth)acrylate, poly(ethylene glycol)s have been used to prepare polymeric scaffolds. Synthetic macromolecules provide sufficient mechanical properties and structural integrity; however, their often poor degradation and capacity to interact with cells without their further modification or functionalization limit their applications as biomaterials [2,3,4,5,6]. On the other hand, natural polymers are an attractive biomaterial alternative, because they offer tissue-appropriate physical and chemical properties that govern and regulate specific cell proliferation and differentiation [7]. The natural polymers, alginate and gelatin, due to their outstanding properties in terms of biodegradability, non-toxicity, non-immunogenicity and chelating ability, are widely used in a variety of biomedical applications, including wound dressings, cartilage and bone tissue engineering, dental impression materials, and as cells delivery systems for tissue repair and regeneration, as well as drug delivery systems [8,9]. Alginate is a polysaccharide extracted from brown seaweeds; considering its excellent biocompatibility, great water/liquid-absorbing capacity and biodegradability, it is recognized as one of the most promising biomaterials [10,11]. However, weak mechanical properties and lack of cellular adhesion limit using alginate as biomaterial, though its combination with other natural polymers, such as gelatin, which can support cell adhesion, migration and proliferation, is a promising approach to form biomaterials [12,13,14]. Gelatin is a natural biopolymer derived from animal collagen; owing to its good biocompatibility and biodegradability and low antigenicity, it is promising scaffolding and drug delivery biomaterial [8]. Some drawbacks (overall mechanical weakness, low stability in the body, etc.) of natural polymers limit their extensive use in preclinical/clinical application in biomedicine [1,15]. Therefore, reinforcing the mechanical strength of natural-based materials is of enormous significance to providing structural properties similar to the specific target tissues. Nowadays, the use of different nanofillers, such as graphene oxide or graphene, has proved to be an efficient method to upgrade the performances of polymeric scaffolding biomaterials [16,17]. Graphene oxide (GO) is the most promising type of nanofiller that greatly improves the mechanical properties of materials applicable in many different fields, including biomedicine and pharmacy [18,19]. The use of graphene oxide in the scaffolding biomaterials is significantly growing, as it promotes cells adhesion, proliferation and differentiation [20,21,22,23]. The structure of graphene controls anatomical structure, which gives the cell shape and coherence and thereby promotes cell proliferation [21]. The application of graphene oxide as a single material for in vivo tissue regeneration demonstrated various drawbacks, such as cytotoxicity and oxidative stress, which are health hazards to the cells and tissues [24]. Thus, the designing-coupled application of the natural and/or synthetic macromolecules and graphene/graphene oxide is of paramount importance for advanced development of tailoring biomedical therapy, including tissue engineering and controlled/targeted drug delivery, targeted adsorption and recycling of organic solvents and oils in extreme environments, effective flame retardant and smoke suppression agent for epoxy resin, and novel efficient anticorrosive coating [25,26,27]. The current state-of-the-art in the development of innovative graphene-based hydrogels includes various methods for their preparation, such as characteristic modification by combining mixing and dispersing, freezing and thawing, shaking and sonication or polymerization, providing valuable additives, including crosslinkers and ions and the development of a new technique for the preparation of graphene-loaded hydrogels is of great interest in chemical and materials science [28]. Accordingly, the novelty of this study is the new approach of using the porogen leaching technique to prepare the porous structure of hydrogel and to incorporate nanoparticles of graphene oxide through the hydrogel simultaneously. The proposed technique is the most practical approach to form porous hydrogels; in this study, we used for the first-time gas bobbles obtained by thermal decomposition of porogen (NaHCO_3_) to lift up and evenly disperse nanoparticles of graphene oxide. From the above-mentioned different techniques, the proposed technique is highlighted due to the simplicity in synthesis, inexpensiveness and absence of potentially toxic side products of solvent/chemical reactions and forming equipment.

This study focused on creatively coupling different materials: synthetic monomer (2-hydroxyethyl methacrylate, HEMA), natural polymers (gelatin (G) and alginate (A)) and nanomaterial (graphene oxide, GO) to design and develop the novel, biocompatible, degradable 3D hydrogels with tunable morphology, swelling capacity, porosity, mechanical properties and drug loading and release performances. To evaluate the hydrogels as efficient drug delivery systems, curcumin, as a drug with poor solubility in water and low bioavailability and with a great scientific challenge to its formulation, was used for loading and in vitro release study. The obtained results confirm that the synergistic effect of the multi-components produces the new hydrogels, which exhibit a novel structure organization and new morphological, mechanical and biological features which significantly develop the extent of their uses in biomedicine, tissue engineering and drug delivery.

## 2. Results and Discussion

### 2.1. Hydrogel Synthesis

**Scheme 1:**
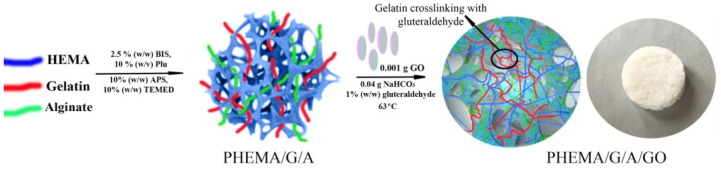
The path of the synthesis PHEMA/G/GO, PHEMA/50G/50A/GO and PHEMA/A/GO hydrogels.

In this study, the novel 2-hydroxyethyl methacrylate/gelatin/alginate hydrogels infused by nanocolloidal graphene oxide was engineered and evaluated as biomaterials that can serve as polymeric scaffolding biomaterials for tissue engineering applications and controlled drug delivery. The path of the synthesis PHEMA/G/A/GO hydrogels is shown in Scheme 1. Specifically, the 2-hydroxyethyl methacrylate was formed mechanically strong and superporous polymeric network by the free-radical polymerization in the presence of polymerization agents (initiator-ammonium persulfate (APS), crosslinking agent-*N*,*N*′-methylenebis(acrylamide) (BIS), and activator-*N*,*N*,*N*′,*N*′-tetramethylene diamine (TEMED)). Gelatin crosslinked by glutaraldehyde created a supplementary polymeric network, while the non-crosslinking chains of alginate were integrated through the polymeric networks. The porosity of the hybrids and distributing of the nanoparticles of GO equally through the polymeric network were accomplished by CO_2_ gas bubbles obtained by thermal decay of NaHCO_3_. In the research, the porogen leaching method was used for the synthesis of the superporous structure only. Herein, the CO_2_ gas bubbles technique was used for the first time to lift up and distribute GO nanoparticles equally through the polymeric network and to simultaneously form a porous structure. The proposed reaction of polymerization 2-hydroxyethyl methacrylate/gelatin/alginate/graphene oxide hydrogels is simple, quick and not difficult to manipulate.

### 2.2. Structural Characteristics of PHEMA/G/A/GO Hydrogels

**Figure 1:**
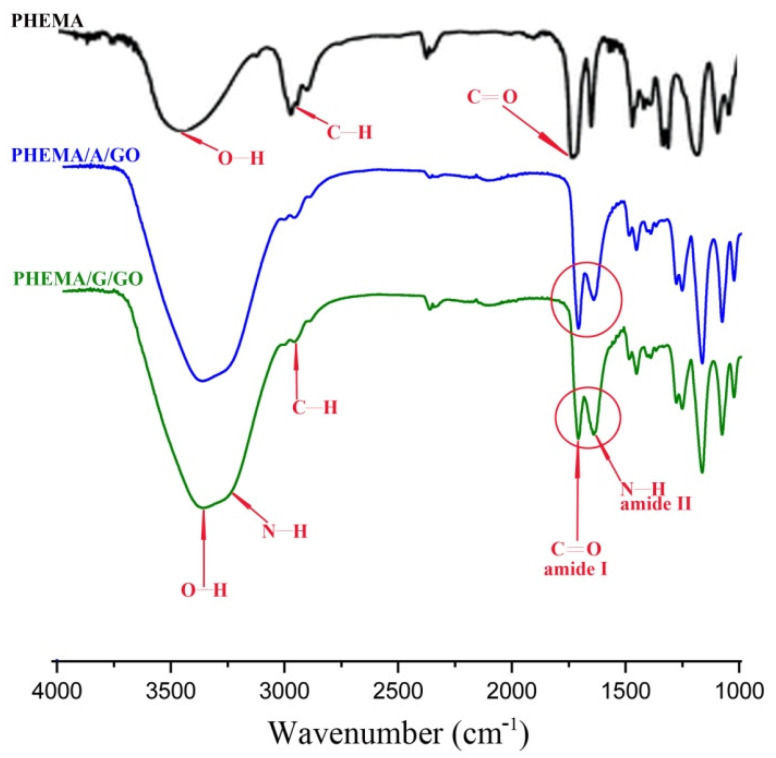
FTIR spectra of PHEMA, PHEMA/A/GO and PHEMA/G/GO hydrogels.

The FTIR spectra of PHEMA, PHEMA/A/GO, and PHEMA/G/GO samples are shown in Figure 1. The PHEMA spectrum showed: O–H stretching vibration at approximately 3431 cm^−1^, strong C=O vibration at 1716 cm^−1^ and C–H stretching vibrations at 2929 cm^−1^ and 2885 cm^−1^ [29]. FT-IR spectra of the PHEMA/A/GO and PHEMA/G/GO materials exhibited above listed PHEMA signals, gelatin (N–H stretching vibration around 3354 cm^−1^, C–H stretching at 3003 cm^−1^, C=O stretching at 1701 cm^−1^ for amide I, N–H definition at 1630 cm^−1^ for the amide II ) and alginate (1278 cm^−1^ C–O stretching, 1160 cm^−1^ C–C stretching, 1017 cm^−1^ C–O–C stretching) vibrations and the type and ratio of the component influenced on the intensity of the peaks [30,31]. The O–H stretching vibration at 3431 cm^−1^ in the spectrum of PHEMA shifted to 3304 cm^−1^ in the spectra of PHEMA/A/GO and PHEMA/G/GO, indicating the presence of oxygen/functional groups of GO [32].

### 2.3. Swelling Study

**Figure 2:**
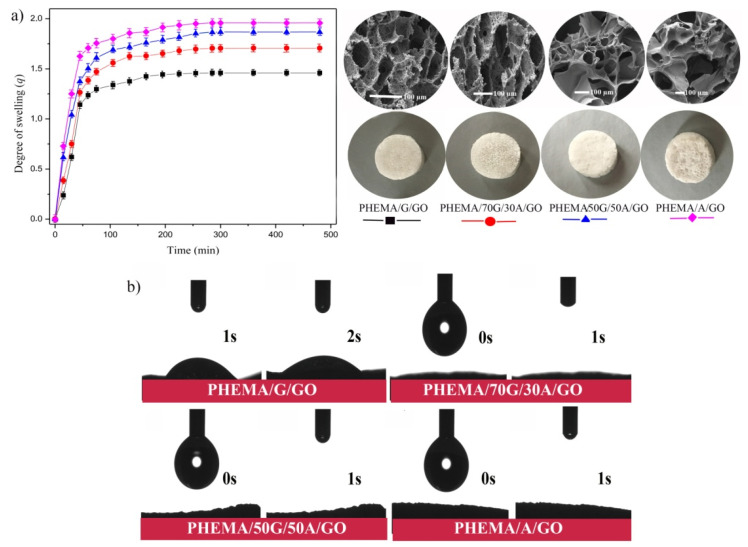
Values of: (**a**) degree of swelling of PHEMA/G/GO, PHEMA/70G/30A/GO, PHEMA50G/50A/GO and PHEMA/A/GO samples (average values from triplicate measurements) versus time; (**b**) water surface contact angle measurements of hydrogels.

The capacity of the biomaterials to uptake water and to support the moving of nutrients is an essential element in the design of scaffolding biomaterials [33]. Hence, the swelling capacity of the synthesized hydrogels was examined in a phosphate buffer solution of pH 7.4, at 37 °C (simulated physiological conditions). The swelling results of the hydrogels were presented in Figure 2 as the degree of swelling (*q*) versus time. As shown from the obtained results, all the hydrogel’s composition reached equilibrium after 4 h of immersion starting time. The PHEMA/A/GO is approximately 1.5 times more hydrophilic compared to PHEMA/G/GO, as reflected from the degree of swelling. This confirms that the swelling capacity of the hydrogels depends on their composition. Thus, introducing, as well as increasing, the content of alginate into the composition of the hydrogel increases the absorbing capacity of the samples. It is well known that the ability of the material to absorb water strongly depends on its hydrophilicity and microstructure, as well as on values of degree of cross-linking [34,35]. This agrees with the obtained results since the materials containing alginate with the lowest level of cross-linking revealed the highest swelling degree. All synthesized hydrogels are capable of absorbing large amounts of water, which is significant because of possible uses in the field of tissue engineering since the materials capable of absorbing plenty of water possess a higher level of mimicry with the natural aqueous environment of cells [36].

### 2.4. Water Contact Angle Study

Numerous studies demonstrate that in vivo tissue compatibility, as well as in vivo functionality and safety of many medical devices (including drug release systems), may be modified by varying surface characteristics, including hydrophilicity. The surface hydrophilicity of the synthesized hydrogels was investigated by water surface contact angle measurements. It is obvious from Figure 2b that all hydrogels are fully hydrophilic (measurements at 0 and 1 s). Water moistens their surfaces and drops currently vanish after application on the hydrogel’s surface, confirming PHEMA/G/A/GO as promising candidates for scaffolding biomaterials of various types of cells.

### 2.5. Morphology

**Figure 3:**
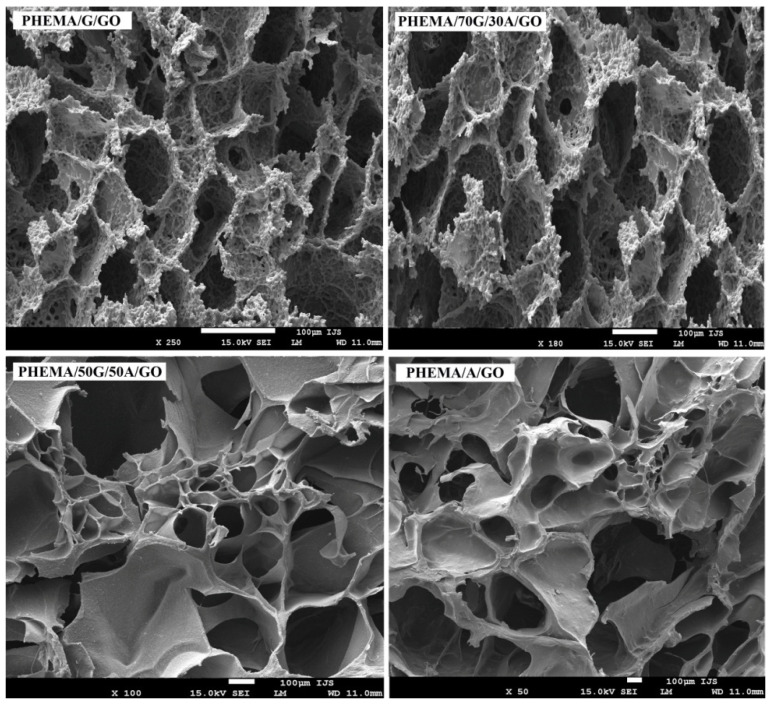
SEM-Micrographs of a cross-section of PHEMA/G/GO, PHEMA/70G/30A/GO, PHEMA50G/50A/GO and PHEMA/A/GO hydrogels.

An ideal scaffolding biomaterial should meet certain criteria, such as providing a suitable 3D porous structure with interconnected pores to promote cell adhesion and growth, as well as the transport of oxygen, nutrient and waste [37]. The porous structure is also crucial for the efficient delivery of the different types of drugs. For this reason, morphological structure of the biomaterials must be ideally engineered and controlled. The morphology of the xerogels cross-section was examined using SEM, and the micrographs are presented in Figure 3.

The micrographs indicated the presence of well-interconnected porous morphology in all samples. The micrographs of the hydrogels containing alginate showed a more asymmetrical and disoriented porous structure, whereas the PHEMA/G/GO hydrogel exhibited a fine porous structure with a fairly uniform distribution. Withal, the pore size increased with the increasing content of alginate into hydrogels. The more porous morphology with the largest interconnected pores of the hydrogels based on alginate can be related to their higher swelling capacity compared with hydrogels without alginate, owing to the porosity and pore size, depending on the water content of swelling hydrogels, before the freeze-drying process. The results of the analysis of hydrogel’s morphology suggested all hydrogels as suitable biological substitutes in tissue engineering applications. The presence of micropores and inter-connectivity in the hydrogels supported a role for allowing cells infiltration, nutrient transport and metabolic waste elimination within the polymeric network.

### 2.6. Porosity

The degree of porosity in the scaffolding materials is crucial to successfully support cell adhesion and growth, and it should match the porosity value for natural human tissue [38]. Due to the porosity having a substantial effect on the mechanical properties of the scaffolding biomaterials, it is crucial to make a balance between mechanical properties and porosity of the biomaterial to satisfy its final application. Obtained results of the porosity measurements are presented in Table 1, showing that the chemical hydrogel’s composition control porosity. It is obvious that increasing the content of alginate in the hydrogels increases the degree of their porosity from 53 to 76% (Table 1). The obtained trend can be explained by the higher swelling capacity of the hydrogels based on alginate, which possesses a larger content of water, resulting in larger pore formation from ice during the freeze-drying process. An additional explanation of the highest porosity of the hydrogels based on alginate is their lowest crosslinking degree compared to hydrogels based on gelatin, which is additionally crosslinked by glutaraldehyde.

### 2.7. Mechanical Properties of the Hybrid Hydrogels

To serve its function as a biomechanical structure scaffolding, biomaterial should match the mechanical features of target natural tissue [20,39]. The elastic modulus of the scaffolding materials is one of the most important parameters used to evaluate their potential for biomedical application. Thus, the biomechanical properties of the PHEMA/G/A/GO hydrogels were analyzed by values of Young’s modulus (Table 1).

The values of Young’s modulus of PHEMA/G/A/GO hydrogels depend on chemical composition. The values of Young’s modulus decreased with the increasing content of alginate into the hydrogels. The hydrogels containing alginate (PHEMA/50G/50A/GO and PHEMA/A/GO) show lower values of Young’s modulus (2.68 and 1.69 MPa), owing to greater porosity than the hydrogels based on gelatin PHEMA/G/GO (4.78 MPa). It can be concluded that the mechanical properties of the PHEMA/G/A/GO hydrogels can be tailored by simply varying the chemical composition of the hydrogels.

### 2.8. In Vitro Degradation Rate of the Hydrogels

Adequate degradation performance of biomaterials is also an essential factor for their applications in tissue engineering and drug delivery systems. Herein, the possibility of improving the degradability of HEMA-based hydrogels was presented due to poly(2-hydroxyethyl methacrylate) being biocompatible but not biodegradable. The degradation rate of the PHEMA/G/A/GO hydrogels was evaluated in vitro by immersing samples into phosphate buffer solution (pH 7.40 at 37 °C simulates physiological conditions) during three and six months; results are shown in Table 1 as a percentage of their weight loss. It is obvious that natural polymers introduce the biodegradability of HEMA-containing hydrogels, the degradation range after three months is 15–22% and after six months 41–44%. The chemical composition of the hydrogels influences their degradation behavior, as was expected. Increasing the content of gelatin decreases the degradation of the hydrogel, suggesting that the level of crosslinking is certainly a crucial factor for designing degradation of the hydrogels [40].

### 2.9. In Vitro Cell Viability, MTT

**Figure 4:**
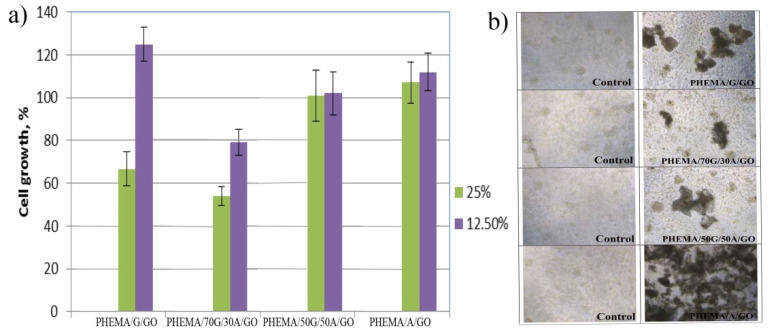
Effect of (**a**) 12.5, and 25% PHEMA/G/GO, PHEMA/70G/30A/GO, PHEMA/50G/50A/GO and PHEMA/A/GO extracts on MRC5 cell viability (Bonferroni test, *p* = 0.05); (**b**) hybrids supplied directly to MRC5 cells.

Cytocompatibility is a mandatory attribute to evaluate the compatibility of biomaterials used for tissue engineering and medical devices, including drug delivery systems. In vitro cell viability of the PHEMA/G/A/GO hydrogels was analyzed by healthy human fibroblasts (MRC5) treated (48 h treatment) with 25 and 12.5% (*v*/*v*) material extract, as shown in Figure 4. The extracts of PHEMA/G/A/GO hydrogels were obtained after extended shaking and immersion in RPMI medium at 37 °C for 3 days. The extracts of PHEMA/50G/50A/GO and PHEMA/A/GO did not show in vitro cytotoxicity, doses of 12.50% PHEMA/G/A/GO extracts influenced higher proliferation in MRC5 cells compared to control that was 100% (Figure 4a). The stimulative effect on MRC5 cell proliferation was the most noticeable by 12.5% extract of PHEMA/G/GO hydrogel. All PHEMA/G/A/GO hydrogels applied to MRC5 cells supported the accumulation of cells on its surface (Figure 4b), indicating nontoxicity of PHEMA/G/A/GO to human fibroblasts.

### 2.10. Drug Loaded and Entrapment Efficiency

Due to the biocompatibility of the PHEMA/G/A/GO hydrogels having been confirmed, a further aim of this study is to confirm their applicability as systems for loading and release of active agents where curcumin was chosen as a model drug. Curcumin is a natural polyphenol with numerous biological effects, such as antioxidant, antibacterial, antifungal, anti-inflammatory, and antiviral; however, its clinical applications are limited due to poor solubility in water and low bioavailability [41]. The development of the pharmaceutical curcumin formulation to overcome the previously mentioned obstacles is a great challenge in the field of biomaterials science [42,43]. In the literature, available data of human clinical trials for curcumin was used for: colorectal cancer 0.036–0.18 g/day during 4 months, pancreatic cancer 1.5 g/day during 6 weeks, breast cancer 6 g/day during 7 days every 3 weeks, prostate cancer 0.1 g/day during 6 months, osteoarthritis 0.2 g/day during 3 months [44,45,46,47,48]. Based on repeated studies, curcumin has been granted an acceptable daily intake level of 0.1–3 mg/kg-BW by the Joint FAO/WHO Expert Committee on Food Additives [49]. The results obtained for curcumin loading (DL) and entrapment efficiency (EE) of the PHEMA/G/A/GO hydrogels are presented in Table 1 and revealed that the chemical composition of the hydrogels influenced DL and EE values. PHEMA/G/GO loaded a lesser quantity of curcumin (47.99 mg drug/g hydrogel) owing to its lower swelling capacity compared to PHEMA/A/GO hydrogel, which loads the higher curcumin quantity (49.86 mg drug/g hydrogel). In this study, 10 wt% of curcumin was used concerning PHEMA/G/A/GO hydrogels and, according to obtained results for drug loading, all samples satisfy the dosage range for colorectal cancer therapy. The results obtained for the entrapment efficiency of curcumin showed that all PHEMA/G/A/GO hydrogels loaded curcumin with entrapment efficiency above 95%. PHEMA/A/GO hydrogels showed 99.72% entrapment efficiency of curcumin while PHEMA/G/GO showed 95.97%. Besides obtained DL and EE data it should be pointed out that hydrophobic active agent could be efficiently loaded by PHEMA/G/A/GO and an optimal drug formulation can be designed by tuning hydrogel’s composition.

### 2.11. Release Behavior of the Hydrogels

**Figure 5:**
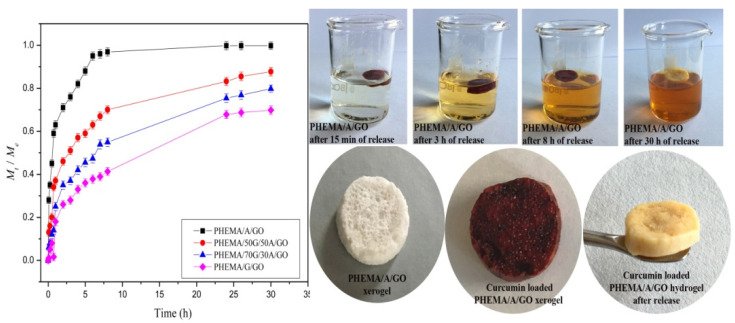
Curcumin release profiles from the hydrogels and photographs of unloaded, curcumin loaded and hybrid after the release of curcumin.

To examine the curcumin release potential from PHEMA/G/A/GO hydrogels, in vitro curcumin release was investigated in a buffer solution of pH 8.0, at 37 °C. The results obtained are presented in Figure 5 and revealed the dependence of curcumin release rate on hydrogel’s composition. Rising alginate content in the samples increases the curcumin release rate, owing to their better swelling capacity compared to PHEMA/G/GO and PHEMA/70G/30A/GO hydrogels. Additionally, the amount of loaded curcumin also influences the release rate because the drug diffusivity depends on the different drug loading levels, thus the hydrogels loaded with higher content of curcumin (PHEMA/A/GO, PHEMA/30G/70A/GO) exhibited a faster release rate.

### 2.12. Analysis of the Drug Transport Mechanism

**Figure 6:**
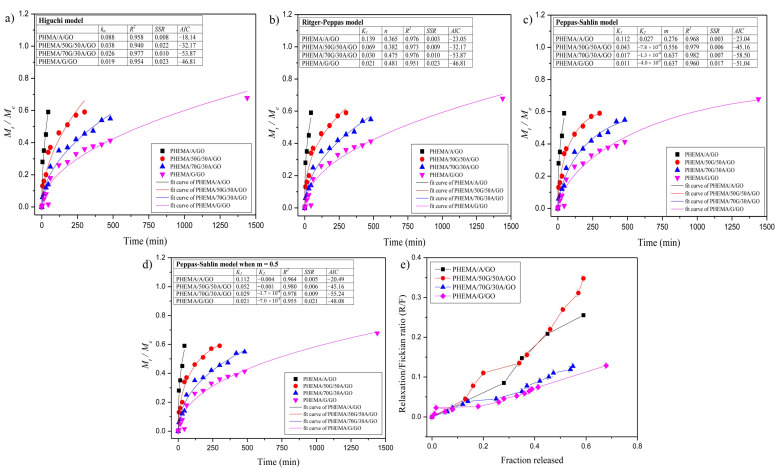
Obtained experimental data of curcumin release from the hybrids fitted by: (**a**) Higuchi; (**b**) Ritger–Peppas; (**c**) Peppas–Sahlin; (**d**) Peppas–Sahlin model when *m* = 0.5; (**e**) the ratio R/F versus the fraction of curcumin released from the hybrid hydrogels.

To develop the kinetics of curcumin release from PHEMA/G/A/GO hydrogels, five different kinetics models were used [50,51,52,53,54,55]. The first 60% of in vitro obtained curcumin release data for PHEMA/G/A/GO hydrogels was fitted; calculated parameters are presented in Figure 6. Experimental data were statistically analyzed by the Akaike information criterion (AIC) values and revealed that Peppas–Sahlin (Figure 6b) and Peppas–Sahlin when *m* = 0.5 (Figure 6d) models best describe release phenomena from curcumin/PHEMA/G/A/GO systems.

The ratio between the relaxation (R) and Fickian (F) contributions (R/F) during the curcumin release was calculated by Peppas–Sahlin equation and shown in Figure 6e. PHEMA/G/GO hydrogel shows a lower R/F ratio than hydrogels containing alginate, which confirms the anomalous curcumin transport mechanism from PHEMA/70G/30A/GO, PHEMA/50G/50A/GO and PHEMA/A/GO, as well as the significance of the relaxation contribution for curcumin release, which is more conspicuous in these hydrogels compared to the PHEMA/G/GO hydrogel.

## 3. Conclusions

The novel, degradable and biocompatible 2-hydroxyethyl methacrylate/gelatin/alginate hydrogels infused by nanocoloidal graphene oxide were successfully synthesized by the adapted porogen leaching method, which, for the first time, was used to incorporate nanocolloidal graphene oxide through the hydrogel and which simultaneously formed a porous hydrogel structure. To obtain the biomaterials with ideal performances for tissue engineering and active agent delivery application, the hydrogel’s chemical composition was varied and evaluated its effect on the hydrogel’s properties. It was found that the multicomponent PHEMA/G/A/GO biomaterials exhibit porous structure with interconnected pores, tunable porosity to 76%, in vitro degradation behavior upward of 40% after 6 months, good swelling capacity, fully hydrophilic surfaces, suitable biocompatibility on MRC5 cells and curcumin entrapment efficiency above 99%, as well as tunable curcumin release performances. Obtained unique performances of PHEMA/G/A/GO hydrogels support their potential application as a successful alternative in biomedical/tissue engineering and controlled active agents delivery systems.

## 4. Materials and Methods

### 4.1. Materials

The monomer 2-hydroxyethyl methacrylate (HEMA, 99%) and polymers: gelatin from porcine skin (G, Type A) and sodium alginate (A) and nanomaterial graphene oxide (GO, nanocolloids, dispersion in water) were supplied from Sigma-Aldrich, Germany. Cross-linking agents: *N*,*N*′-methylenebis(acrylamide) (BIS) and glutaraldehyde, initiator-ammonium persulfate (APS), activator-*N*,*N*,*N*′,*N*′-tetramethylene diamine (TEMED), foaming stabilizer-Pluronic F-172 were obtained from Sigma-Aldrich, Germany. Foaming agents-sodium bicarbonate (NaHCO_3_) and curcumin (from Curcuma longa) were purchased from Sigma-Aldrich, Germany. RPMI-1640 medium and supplements for cell proliferation, as well as 3-(4,5-dimethylthiazol-2-yl)-2,5-diphenyltetrazolium bromide (MTT) reduction assay components were purchased from Sigma-Aldrich. Distilled water was used for all polymerizations and preparations of buffer solution.

### 4.2. Hydrogel Synthesis

PHEMA/G/A/GO hydrogels were synthesized using the adapted porogen leaching technique. To design the hydrogel with the best performance for scaffolding biomaterials and active agent delivery application, the hydrogel’s chemical composition was varied. Precisely, 2250 µL HEMA, 300 µL 2.5% BIS solution and 300 µL 10% Pluronic solution were added into a test tube and vigorously stirred at 50 °C for 1 h, with adding solutions of gelatin and sodium alginate (Table 2); the reaction mixture was stirred for 45 min. After that, 150 µL 10% APS and 150 µL 10% TEMED were added as initiator and activator and the reaction mixture was heated at 63 °C. Then the mixture of 0.04 g NaHCO_3_ and 0.001 g GO was added quickly to the reaction mixture and vigorously vortexed to distribute CO_2_ gas bubbles, which lift up GO nanoparticles evenly throughout the samples and simultaneously form pores into hydrogels structure. After the reaction, the fresh hydrogels were cut into discs and immersed in 1% glutaraldehyde for 2 h; then the samples were oven-dried to constant mass and immersed in distilled water for 7 days, to remove unreacted chemicals. Water was changed daily. The swollen hydrogels were frozen, freeze-dried and used for further experiments.

### 4.3. Hydrogel Characterization

Fourier transform infrared spectroscopy (FTIR): The chemical structure of PHEMA/G/A/GO xerogels was analysed by Fourier transform infrared spectra recorded on an FT-IR Nicolet 6700 (Thermo-Scientific) diamond crystal spectrometer with attenuated total reflectance (ATR) sampling technique over the wavelength range of 4000–700 cm^−1^.

Scanning electron microscopy (SEM): The morphology of the PHEMA/G/A/GO xerogels was observed using a scanning electron microscope (SEM), JEOL JSM-7600F. The swelled PHEMA/G/A/GO hydrogels were freeze-dried, lyophilized and cut into slices, fixed on a holder using carbon tape, sputtered with gold BAL-TEC SCD 005, and dried in a VC 50 SalvisLab Vacucenter vacuum chamber.

Mechanical testing: Mechanical properties of the PHEMA/G/A/GO hydrogels were analysed using Galdabini Quasar 50, Italy, by applying a uniaxial compression with 100-N load cell at room temperature. The Young’s modulus (*E*) of the hydrogels was calculated from the linear part of the stress/strain curve. Each measurement was repeated three times and the final value of Young’s modulus was given as the average value.

Water contact angle measurements: The static water contact angle measurement was realized by the sessile drop method by putting approximately 1 μL drop of MilliQ water on the hydrogel’s surface. The measurements were performed using Theta Lite-Biolin Scientific Contact angle meter in measuring range from 0 to 180 deg. and accuracy +/−0.1 deg., +/−0.01 mN/m with the camera of 640 × 480 resolution and a maximum measuring speed of 60 fps.

Porosity measurement: Porosity of the PHEMA/G/A/GO hydrogels was calculated by true and bulk density of the hydrogels and density obtained by the Archimedes method, whereas a wetting medium of glycerol was used with a density ρ_0_ = 1.2038 g/cm^3^ [25].

Swelling study: The swelling capacity of the PHEMA/G/A/GO hydrogels was evaluated in phosphate buffer solution pH 7.4, at 37 °C mimicking physiological conditions using gravimetric method [52,53,54]. In vitro degradation studies: Hydrolytic degradation studies were evaluated in vitro by dipping the PHEMA/G/A/GO hydrogels in 10 mL phosphate buffer solution, simulating physiological conditions pH 7.4, at 37 °C, during 3 and 6 months [55].

Cell viability assay: Cytotoxicity/antiproliferative activity was measured using MTT assay by the following procedure: 100 mg of the PHEMA/G/A/GO hydrogels were aseptically ground and incubated in 10 mL of RPMI-1640 medium for 72 h at 37 °C and 180 rpm. Human lung fibroblast (MRC5 cells) were cultured 24 h in RPMI-1640 medium enriched with 10% (*v*/*v*) heat-inactivated fetal bovine serum (FBS), 100 U/mL penicillin and 100 µg/mL streptomycin at 37 °C in a humidified atmosphere in the presence of 5% CO_2_. After incubation, MRC5 cells were exposed to 25% and 12.5% (*v*/*v*) hydrogel extract, as well as to 200 µg/mL of the ground and filtered hydrogel and incubated for a further 48 h. Control cultures were treated with growth medium and blank wells also contained growth medium (200 µL). Cells proliferation was calculated by measuring the absorbance at 540 nm on Tecan Infinite 200 Pro multiple reader (Tecan Group, Mannedorf, Switzerland). The measurements were performed in triplicates and the results were presented as a percentage of the control that was arbitrarily set to 100%.

Drug loading and entrapment efficiency, in vitro controlled release study and analysis of the active agent transport mechanism: Curcumin was loaded into the hydrogels using the swelling-diffusion method; curcumin was firstly dissolved in phosphate buffer solution pH 8.00 and then the xerogels were swollen in 5 mL of curcumin solution to reach an equilibrium state [50]. Curcumin release from the PHEMA/G/A/GO hydrogels was conducted in vitro in a basket stirrer containing 15 mL of release medium (phosphate buffer pH 8.0) at 37 ± 0.5 °C. The amount of the released curcumin was measured by taking absorbance of the solution containing released curcumin at regular time intervals using UV/Vis spectrophotometer (Shimadzu UV/Vis Spectrophotometer UV-1800) at λ_max_ value of 430 nm. To evaluate the release kinetics, the first 60% of the in vitro-obtained curcumin release data of the PHEMA/G/A/GO hydrogels was fitted to the different kinetic models [50,56].

## Figures and Tables

**Table 1:** Obtained results of porosity, Young’s modulus, weight loss (%) after 3 and 6 months, drug loading and entrapment efficiency of the hybrid hydrogels.

Hydrogel	Porosity (%)	Young’s Modulus (MPa)	Weight Loss (%) after 3 and 6 Months	Drug Loading (mg Drug/g Hydrogel)	Entrapment Efficiency (%)
PHEMA/G/GO	52.80	4.78	14.88; 40.91	47.99	95.97
PHEMA/70G/30A/GO	70.49	4.34	16.98; 41.58	48.11	96.21
PHEMA/50G/50A/GO	71.40	2.68	18.01; 42.97	48.82	97.63
PHEMA/A/GO	76.36	1.69	22.04; 44.29	49.86	99.72

**Table 2:** Composition of the gelatin and sodium alginate solutions and designation of the hydrogels.

Hydrogel	10% Gelatin (G) (µL)	10% Sodium Alginate (A) (µL)
PHEMA/G/GO	2250	0
PHEMA/70G/30A/GO	1575	675
PHEMA/50G/50A/GO	1125	1125
PHEMA/A/GO	0	2250

## Data Availability

The data presented in this study are available on request from the corresponding author.
